# Response: Commentary: Dog Stick Chewing: An Overlooked Instance of Tool Use?

**DOI:** 10.3389/fpsyg.2021.757526

**Published:** 2021-09-24

**Authors:** James Brooks, Shinya Yamamoto

**Affiliations:** ^1^Wildlife Research Center, Kyoto University, Kyoto, Japan; ^2^Insittute for Advanced Study, Kyoto University, Kyoto, Japan

**Keywords:** dog cognition, tool use, cognitive evolution, comparative cognition, teething, object manipulation

Iotchev (2021) provides critical commentary to our recent suggestion that dog stick chewing may represent a previously overlooked form of tool use (Brooks and Yamamoto, 2021). We appreciate the engagement in our hypothesis, and agree with his final constructive paragraphs emphasizing the importance of creative and novel behavioral studies alongside neuroscientific research in better understanding such behaviors. However, we believe many of his more critical points rest on misunderstandings of both our initial paper and the existing tool use literature.

Most significantly, while we do emphasize that tool use as such can occur without sophisticated cognitive background, we are not the first to suggest this. This is in line with much previous research rather than in opposition, for example recently in Call (2013), Hansell and Ruxton (2008), Matsuzawa (2001), von Bayern et al. (2020), and in earlier work such as Hall (1963) and Beck (1980). We are not removing higher order cognition and understanding from tool use, but instead Iotchev is inserting it. Tool use typically describes a behavior rather than a mental process. While definitions do not always agree on a precise singular characterization, most focus on how an object is used, its effects, and the functionality of the tool rather than Iotchev's emphasis on the role of self-awareness, mental representations, and even consciousness. Consider Shumaker et al.'s 2011 definition which is arguably the most widely used and the definition on which we rest the majority of our argument: “The external employment of an unattached or manipulable attached environmental object to alter more efficiently the form, position, or condition of another object, another organism, or the user itself, when the user holds and directly manipulates the tool during or prior to use and is responsible for the proper and effective orientation of the tool.” There is no reference to any particular cognitive process. Indeed, in the same book Shumaker et al. (2011) put as #2 in their list of animal tool use myths that “Tool use is intelligent” and give extended treatment to debunking this myth. We are not removing these elements from existing definitions to widen their scope, but instead Iotchev is adding them in an attempt to narrow what can be viewed as tool use.

Iotchev (2021) further criticizes our proposed spectrum of cognition behind tool use, or continuum as he calls it, on grounds that it (1) cannot provide meaningful transition points which he claims it promises and (2) does not provide any specificity about which behaviors should be considered ancestral to tool use. Again, we believe this lies on a misunderstanding of our point. Regarding the first point, we do not intend to promise meaningful transition points at all. While we identify broad regions, these are not suggested as hard lines, but lie along a continuous spectrum. It is Iotchev who is suggesting a hard transition, a binary between tool use and non-tool use, while we attempt to recognize the range of complexity and point to some general areas on the spectrum. Regarding his second point about “behaviors ancestral to tool use,” we make no claims. In our previous paper we suggest dog stick chewing can be considered tool use in itself, not simply ancestral. The proposed spectrum describes the range of possible cognitive complexity behind tool use behaviors rather than describing ancestral behaviors.

Still, we certainly recognize the importance of studying higher level abilities and their role in tool use. We firmly agree with Iotchev that in some cases, tool use can test the upper limits of animal cognition. We suggest not that these should be ignored or viewed as identical to forms of tool use with less complex cognitive background, but see the variation and continuity that exists as a meaningful and promising area of study. Why some species should be equipped with relatively hardwired, inflexible forms of tool use, while others can use planning, spatial reasoning, and detailed mental representations of their surroundings, is an engaging question which suggests new areas of investigation into the evolution of higher order cognition. The neuroscientific avenue described by Iotchev furthers this view. Identifying the neural correlates of tool use with varying complexity, and between different species in similar paradigms, can shed light on the evolutionary and psychological background that enables some of the most complex and interesting forms of cognition. The spectrum we begin to describe can work together with Iotchev's suggestions.

We believe the fundamental disconnect between the views of our previous paper and Iotchev's reply is whether to view tool use as a way of interacting with an external object or as one specific mental process. We follow previous tool use literature and definitions in identifying dog stick chewing with the behavioral definitions of how an animal may interact with the environment (for example Beck, 1980 explicitly rejects cognitive processes from his definition). The specific mental process involved in the most complex forms of tool use, including physical reasoning and object representations, is perhaps a topic worthwhile of being identified for itself. Still, it should not be confused with the behavioral outcome by being called tool use. Many of Iotchev's points rest on viewing “tool use” as a singular internal cognitive process, as opposed to the interactions with the environment they can in some cases produce. In our previous paper we emphasized that tool use can occur as a result of a range of mental processes, and as clearly put by Call (2013) “…tool use is a very broad functional category that includes very different examples whose cognitive substrate may differ substantially between and within species.”

In conclusion, we agree with many of Iotchev's points and sincerely thank him for writing his critique. We believe these issues rest on differing understandings of both tool use as an area of study and the points we intended to make in our previous paper. Recognizing the divide between tool use as a set of behaviors involving interacting with an external object in some specified ways, and the higher order mental process (involving representations, physical reasoning, and intentionality) involved in the most complex forms of tool use, will be important in acknowledging the value of research on both sides. These positions are not in opposition, but should work together to understand the cognitive infrastructure and evolution of tool use and object manipulation across the range of complexity in which they exist.

## Author Contributions

JB drafted the original manuscript. SY provided critical revisions. Both authors contributed to the ideas in this paper.

## Funding

This research was supported by the JSPS Grant-in-Aid for Scientific Research KAKENHI #21J21123 to JB and #19H00629 to SY.

## Conflict of Interest

The authors declare that the research was conducted in the absence of any commercial or financial relationships that could be construed as a potential conflict of interest.

## Publisher's Note

All claims expressed in this article are solely those of the authors and do not necessarily represent those of their affiliated organizations, or those of the publisher, the editors and the reviewers. Any product that may be evaluated in this article, or claim that may be made by its manufacturer, is not guaranteed or endorsed by the publisher.
